# Quantitative assessment of the influence of EPHX1 gene polymorphisms and cancer risk: a meta-analysis with 94,213 subjects

**DOI:** 10.1186/s13046-014-0082-9

**Published:** 2014-09-28

**Authors:** Xiaoqin Yang, Yubing Wang, Guiping Wang

**Affiliations:** Department of bioinformatics, School of Life Science and Technology, Tongji University, Shanghai, 200092 People’s Republic of China; Department of Pharmacy, College of Health sciences, Guangzhou Medical University, Guangzhou, 510180 People’s Republic of China

**Keywords:** EPHX1 polymorphisms, Meta-analysis, Cancer risk

## Abstract

**Purpose:**

Previous studies investigating the association between EPHX1 polymorphisms (Tyr113His and His139Arg) and cancer risk have yielded inconsistent results. This meta-analysis was performed to derive a more precise estimation of relationship between two EPHX1 polymorphisms and risk of different types of cancer.

**Methods:**

Data were extracted from relevant studies detected by a systematic literature search. Odds ratios (ORs) with 95% confidence intervals (CIs) were calculated to assess the strength of the association between EPHX1 polymorphisms and cancer risk.

**Results:**

This meta-analysis carefully collected 99 studies on these two polymorphisms and cancer risk published up to March 2014, consisting of 45 studies (20,091 cases and 27,396 controls) for Tyr113His and 54 studies (19,437 cases and 27,289 controls) for His139Arg. The results in overall population did not show any significant association between these two polymorphisms and cancer risk for all genetic models. However, EPHX1 Tyr113His homozygote individuals have a significantly increased risk of cancer among Asians (homozygote model: OR =1.46, 95% CI=1.05–2.03; recessive model: OR =1.39, 95% CI =1.10–1.76) and mixed population (homozygote model: OR =1.17, 95% CI =1.02–1.34; recessive model: OR =1.17, 95% CI =1.02–1.33), but not Caucasians.

**Conclusion:**

His/His genotype of EPHX1 Tyr113His polymorphism is a risk factor for developing caner for Asian and mixed population, while no evidence was found for the association between the EPHX1 His139Arg polymorphism and increased cancer risk.

**Electronic supplementary material:**

The online version of this article (doi:10.1186/s13046-014-0082-9) contains supplementary material, which is available to authorized users.

## Background

Xenobiotic catalytic pathway is an important defense mechanism against carcinogenesis [1]. As a critical biotransformation enzyme of this pathway, microsomal epoxide hydrolase (EPHX1) plays a key role in the detoxification of potential carcinogens from endogenous compounds as well as exogenous chemicals, which ultimately convert them into less toxic metabolites [2-5].

The EPHX1 gene is located on chromosome 1q42 with 9 exons and 8 introns. Functional studies have shown that two common polymorphic sites in the gene affecting EPHX1 enzyme activity. The tyrosine to histidine substitution in exon 3 (Tyr113His, site: T337C, dbSNP: rs1051740) sharply decreases its enzyme activity by nearly 40%, whereas the histidine to arginine substitution in exon 4 (His139Arg, site: A415G, dbSNP: rs2234922) could increase the enzyme activity by approximately 25% [6]. Given the significance of EPHX1 in eliminating carcinogenicity of toxic compounds like epoxides, it could be proposed that these two functional polymorphisms may lead to individual variations of xenobiotic detoxification and further influence susceptibility to chemical carcinogen-induced cancers.

Over the past two decades, a number of studies have been conducted to investigate the relationship between EPHX1 polymorphisms and cancer in different populations. However, the results of these studies are conflicting rather than conclusive. Several previous meta-analyses were flawed in their lack of sufficient data or there were methodological problems. One meta-analysis by Li et al. found that no significant association between EPHX1 polymorphisms (Tyr113His and His139Arg) and increased risk of cancers [7]. However, several studies [8-10] included in this meta-analysis were incorrectly classified according to source of controls, which may lead to an inaccurate result. Some recent studies did not evaluate the deviations from Hardy–Weinberg equilibrium (HWE) in control subjects [11-13], which could bias the estimates of genetic effects in genetic association studies and meta-analysis [14]. Since that date, several more studies have emerged to assess the relationship between the Tyr113His and/or His139Arg polymorphisms of the EPHX1 gene and susceptibility to a variety of cancers. Given the new information, we systematically evaluated the effect of these two polymorphisms on cancer risk in an updated meta-analysis with increased statistical power in order to get a more precise and reliable assessment of the association.

## Materials and methods

### Search strategy

A comprehensive literature search was performed using PubMed database for relevant articles published (last search: March 14, 2014) with the following terms: ((“epoxide hydrolase 1”) OR EPHX1) AND (((polymorphism) OR (SNP)) OR variant)) AND ((((neoplasm) OR cancer) OR carcinoma) OR leukemia). All the references of retrieved articles and supplementary data were checked when key information relevant to the meta-analysis was missing.

### Inclusion criteria

All studies were included if they met the following criteria: (1) case–control study; (2) studies to evaluate the association between EPHX1 gene polymorphisms (Tyr113His and His139Arg) and risk of cancer; (3) sufficient data for estimating an odds ratio (OR) with 95% confidence interval (CI); (4) full-text in English available and (5) more than 100 patients. When the same population was included in several publications, only the most complete one was included in this meta-analysis.

### Data extraction

Data were carefully evaluated and extracted from the eligible studies by two investigators independently according to the inclusion criteria listed above. The following characters were collected from eligible studies: first author’s name, year of publication, ethnicity (categorized as Asian, Caucasian, African, or mixed), source of control groups (population-based [PB], hospital-based [HB], family-based [FB] or unknown), genotype frequency of cases and controls, and the results of Hardy-Weinberg equilibrium (HWE) test. When it came to discrepancy between two investigators, another investigator was invited to discuss and check the data until a consensus was reached.

### Statistical analysis

The departure from the Hardy-Weinberg equilibrium for the control group in each study was assessed with Pearson's goodness-of-fit Chi-square test with 1 degree of freedom by a web-based program (http://ihg.gsf.de/cgi-bin/hw/hwa1.pl) and the violation of HWE was determined with a threshold of p < 0.05. Odds ratios (ORs) with 95% confidence intervals (CIs) were used to assess the strength of association between the EPHX1 gene polymorphisms and cancer susceptibility. Pooled ORs were performed for dominant model (aa + Aa vs. AA, a was for the minor allele and A was for the major allele), recessive model (aa vs. Aa + AA), homozygote comparison (aa vs. AA), heterozygote comparison (Aa vs. AA), and additive model (a vs. A), respectively. Heterogeneity among pooled studies were evaluated by the Chi-square-based Cochran’s Q test [15] and I^2^ statistics [16]. To be more conservative, heterogeneity was considered to be present when the Cochran’s Q-test P-value was less than 0.1, then random-effects model (the DerSimonian and Laird method) [17] was utilized, otherwise, fixed-effects model was used (the Mantel-Haenszel method) [18]. In addition, inconsistency across studies was quantified by means of I^2^ statistic, with I^2^ < 25%, 25-75%, and >75% considered to represent low, moderate and high degree of heterogeneity, respectively [16]. Stratification analyses were performed to test the effects of cancer types, source of control, ethnicity and smoking status, respectively. To explore the source of heterogeneity among the studies of this meta-analysis, a multivariate meta-regression analysis subjected to 10,000 permutations was undertaken to explore the possible sources of heterogeneity. The following study characteristics were included as covariates in the meta-regression analysis: ethnicity, source of control, cancer types. Sensitivity analysis was carried out through omitting individual study in turn to check the consistency of the results. Publication bias was evaluated by visual inspection of the Begg’s funnel plots [19] and the Egger’s linear regression (P < 0.05 was considered a significant publication bias) [20]. All statistical tests were performed with metafor [21] and meta (http://cran.r-project.org/web/packages/meta/) packages of R (version 3.0.1), using two-sided p-values.

This meta-analysis followed the guidelines of the preferred reporting items for systematic reviews and meta-analysis (PRISMA) statement [22] (Additional file 1: Table S1).

## Results

### Study characteristics

**Figure 1:**
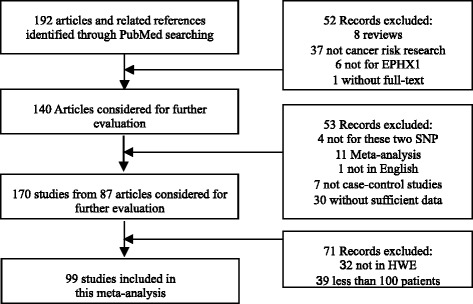
**The flow diagram of the literature search and the study selection.**

The initial literature search through PubMed database yielded 192 published articles. Totally, when reviewed in full-text, 4 were not concerned with Tyr113His or His139Arg polymorphisms in EPHX1 gene, 37 were not cancer risk studies, 1 was not published in English, 1 was not provided in full text, 7 were not case–control studies, 30 were no usable reported data, and 19 were meta-analysis or reviews; all these publications were excluded. Among the remaining 87 articles, studies presented separate OR by different polymorphisms, cancer types or ethnicity, and each of them was considered separately for pooling analysis. Furthermore, 32 studies not in HWE and 39 with less 100 patients were also deleted. Hence, 45 studies [8-10,23-63] for Tyr113His polymorphism (20,091 cases and 27,396 controls) and 54 studies [8-10,24-26,28,29,32,34-45,47-51,53,55-61,63-81] for His139Arg polymorphism (19,437 cases and 27,289 controls) were included eventually. Genotype distributions in the controls of all selected studies are in agreement with HWE. The flow of study selection was shown in Figure 1, and the main characteristics of eligible studies were summarized (Additional file 2: Table S2 and Additional file 3: Table S3).

### Quantitative synthesis

The pooled results of meta-analysis for the association between EPHX1 polymorphisms (Tyr113His and His139Arg) and cancer susceptibility are shown in Tables 1 and 2. Heterogeneity across studies must be considered because it may affect the strengths of the meta-analysis. Significant heterogeneity was observed in some comparisons for both EPHX1 Tyr113His and His139Arg polymorphisms. Thus, random-effect model was used when heterogeneity identified.

**Table 1:** **Overall and stratified meta-analyses of the association between the EPHX1 Tyr113His polymorphism and cancer risk**

**Variables**	**No** ^**a**^	**Case/control**	**Homozygote comparison**			**Heterozygote comparison**			**Dominant model**			**Recessive model**			**Additive model**		
			**OR (95% CI)**	**P**	**P** ^**b**^ **(I** ^**2**^ **)**	**OR (95% CI)**	**P**	**P** ^**b**^ **(I** ^**2**^ **)**	**OR (95% CI)**	**P**	**P** ^**b**^ **(I** ^**2**^ **)**	**OR (95% CI)**	**P**	**P** ^**b**^ **(I** ^**2**^ **)**	**OR (95% CI)**	**P**	**P** ^**b**^ **(I** ^**2**^ **)**
**Total**	45	20091/27396	1.05(0.95 ~ 1.16)	0.37	0.00(50.8)	0.94(0.88 ~ 1.01)	0.08	0.0(54.0)	0.96(0.90 ~ 1.03)	0.26	0.0(56.8)	1.08(0.99 ~ 1.18)	0.08	0.0(37.4)	1.00(0.95 ~ 1.05)	0.94	0.0(57.7)
**Cancer type**																	
Blood	7	2419/2319	1.05(0.76 ~ 1.46)	0.76	0.03(56.1)	0.89(0.73 ~ 1.01)	0.24	0.06(49.8)	0.92(0.75 ~ 1.13)	0.42	0.02(60.3)	**1.17(0.96 ~ 1.41)**	**0.12**	**0.14(37.3)**	0.97(0.82 ~ 1.15)	0.76	0.01(65.9)
Prostate	3	1706/1192	1.54(0.57 ~ 4.16)	0.86	0.00(88.9)	1.31(0.82 ~ 2.01)	0.26	0.00(79.7)	1.39(0.80 ~ 2.41)	0.24	0.00(87.1)	1.29(0.62 ~ 2.65)	0.50	0.0(82.2)	1.27(0.81 ~ 1.97)	0.30	0.00(89.7)
Esophageal	3	593/1086	**1.09(0.78 ~ 1.51)**	**0.63**	**0.19(40.5)**	1.01(0.52 ~ 1.93)	0.99	0.00(86.0)	1.08(0.63 ~ 1.84)	0.79	0.00(81.6)	**1.16(0.85 ~ 1.58)**	**0.34**	**0.20(38.4)**	1.09(0.84 ~ 1.41)	0.51	0.07(61.9)
Colorectal	9	5512/6787	**0.98(0.86 ~ 1.11)**	**0.75**	**0.85(0.0)**	**1.00(0.93 ~ 1.08)**	**0.98**	**0.79(0.0)**	**0.99(0.93 ~ 1.07)**	**0.98**	**0.80(0.0)**	**0.98(0.87 ~ 1.11)**	**0.76**	**0.87(0.0)**	**1.00(0.94 ~ 1.05)**	**0.87**	**0.84(0.0)**
Other	7	2425/2872	1.20(0.90 ~ 1.59)	0.22	0.06(50.4)	1.00(0.81 ~ 1.24)	0.99	0.01(66.7)	1.04(0.84 ~ 1.29)	0.74	0.00(69.4)	**1.18(0.98 ~ 1.42)**	**0.08**	**0.40(2.8)**	1.06(0.91 ~ 1.23)	0.45	0.01(65.5)
Lung	9	2065/5429	0.80(0.57 ~ 1.12)	0.20	0.00(62.5)	0.80(0.65 ~ 0.98)	0.03	0.00(65.0)	0.81(0.68 ~ 0.98)	0.03	0.01(62.5)	0.91(0.66 ~ 1.25)	0.57	0.01(61.7)	0.87(0.75 ~ 1.01)	0.08	0.0(65.5)
Head and neck	3	825/821	**1.04(0.74 ~ 1.47)**	**0.81**	**0.98(0.0)**	**0.87(0.71 ~ 1.07)**	**0.19**	**0.82(0.0)**	**0.90(0.74 ~ 1.10)**	**0.30**	**0.85(0.0)**	**1.11(0.80 ~ 1.54)**	**0.53**	**0.99(0.0)**	**0.96(0.83 ~ 1.12)**	**0.61**	**0.91(0.0)**
Breast cancer	4	4546/6890	**1.02(0.90 ~ 1.17)**	**0.73**	**0.15(43.0)**	**0.99(0.91 ~ 1.07)**	**0.73**	**0.60(0.0)**	**0.99(0.92 ~ 1.07)**	**0.35**	**0.99(0.0)**	1.14(0.89 ~ 1.46)	0.31	0.05(62.6)	**1.00(0.97 ~ 1.03)**	**0.93**	**0.68(0.0)**
**Source of control**																	
PB	31	16035/23111	1.03(0.93 ~ 1.14)	0.54	0.06(29.7)	0.97(0.92 ~ 1.03)	0.28	0.10(25.1)	0.98(0.93 ~ 1.04)	0.45	0.06(30.4)	**1.03(0.96 ~ 1.11)**	**0.38**	**0.14(21.9)**	1.00(0.95 ~ 1.04)	0.91	0.03(34.7)
HB	11	3517/3627	0.94(0.77 ~ 1.15)	0.56	0.08(40.9)	0.81(0.69 ~ 0.96)	0.01	0.00(59.4)	0.85(0.74 ~ 0.98)	0.03	0.03(49.4)	1.05(0.86 ~ 1.27)	0.66	0.05(45.7)	0.93(0.84 ~ 1.03)	0.16	0.05(45.3)
**Ethnicity**																	
Caucasian	26	11757/18447	**0.94(0.87 ~ 1.03)**	**0.17**	**0.29(11.7)**	**0.93(0.87 ~ 1.00)**	0.04	0.09(28.7)	0.93(0.87 ~ 1.00)	0.04	0.06(31.7)	**0.97(0.89 ~ 1.05)**	**0.45**	**0.49(0.0)**	0.96(0.91 ~ 1.00)	0.07	0.08(29.6)
Mixed	7	5645/5502	**1.17(1.02 ~ 1.34)**	**0.03**	**0.42(0.7)**	**1.00(0.93 ~ 1.09)**	**0.96**	**0.57(0.0)**	**1.03(0.96 ~ 1.11)**	**0.44**	**0.53(0.0)**	**1.17(1.02 ~ 1.33)**	**0.02**	**0.44(0.0)**	**1.05(0.99 ~ 1.11)**	**0.11**	**0.43(0.0)**
Asian	11	2534/3205	1.46(1.05 ~ 2.03)	0.04	0.00(75.1)	1.04(0.77 ~ 1.40)	0.81	0.00(81.7)	1.16(0.88 ~ 1.53)	0.30	0.00(56.8)	1.39(1.10 ~ 1.76)	0.01	0.01(60.7)	1.19(0.99 ~ 1.42)	0.05	0.0(79.4)
**Smoking status**																	
Smoker	9	1786/2114	**0.99(0.80 ~ 1.23)**	**0.95**	**0.42(1.4)**	0.85(0.66 ~ 1.10)	0.21	0.03(52.1)	0.89(0.71-1.11)	0.30	0.05(47.8)	**1.07(0.87 ~ 1.31)**	**0.56**	**0.43(0.5)**	**0.97(0.88 ~ 1.08)**	**0.60**	**0.13(36.7)**
Non-smoker	8	1357/1947	1.45(0.89 ~ 2.37)	0.14	0.00(71.4)	1.19(0.88 ~ 1.60)	0.27	0.01(63.6)	1.27(0.92 ~ 1.74)	0.14	0.00(71.7)	1.29(0.92~1.82)	0.14	0.05(50.0)	1.23(0.97 ~ 1.57)	0.09	0.00(75.8)

PB: population based; HB: hospital based. *P* ^*b*^: P-values for heterogeneity from Q test; I^2^ refers to the proportion of total variation owing to between-study heterogeneity.

Bold font marks where fixed effect model used.

**Table 2:** **Overall and stratified meta-analyses of the association between the EPHX1 His139Arg polymorphism and cancer risk**

**Variables**	**No** ^**a**^	**Case/control**	**Homozygote comparison**			**Heterozygote comparison**			**Dominant model**			**Recessive model**			**Additive model**		
			**OR (95% CI)**	**P**	**P** ^**b**^ **(I** ^**2**^ **)**	**OR (95% CI)**	**P**	**P** ^**b**^ **(I** ^**2**^ **)**	**OR (95% CI)**	**P**	**P** ^**b**^ **(I** ^**2**^ **)**	**OR (95% CI)**	**P**	**P** ^**b**^ **(I** ^**2**^ **)**	**OR (95% CI)**	**P**	**P** ^**b**^ **(I** ^**2**^ **)**
**Total**	54	19437/27289	1.05(0.93 ~ 1.18)	0.73	0.09(21.3)	0.96(0.91 ~ 1.01)	0.15	0.02(31.5)	0.97(0.92 ~ 1.03)	0.30	0.00(40.1)	**1.02(0.93 ~ 1.13)**	**0.66**	**0.23(12.0)**	0.99(0.94 ~ 1.04)	0.57	0.00(44.9)
**Cancer type**																	
Other	11	3160/4581	**0.94(0.75 ~ 1.20)**	**0.64**	**0.53(0.0)**	**0.97(0.87 ~ 1.10)**	**0.51**	**0.56(0.0)**	**0.97(0.87 ~ 1.06)**	**0.47**	**0.53(0.0)**	**0.96(0.76 ~ 1.21)**	**0.71**	**0.53(0.0)**	**0.97(0.89 ~ 1.05)**	**0.50**	**0.46(0.0)**
Blood	9	2929/3629	**0.87(0.67 ~ 1.13)**	**0.29**	**0.90(0.0)**	**0.90(0.81 ~ 1.00)**	**0.05**	**0.25(21.4)**	**0.90(0.81 ~ 0.99)**	**0.04**	**0.47(0.0)**	**0.90(0.70 ~ 1.17)**	**0.44**	**0.82(0.0)**	**0.91(0.83 ~ 0.99)**	**0.04**	**0.80(0.0)**
Esophageal	3	593/1081	1.30(0.50 ~ 3.38)	0.59	0.07(61.8)	1.02(0.65 ~ 1.62)	0.92	0.03(72.6)	1.06(0.66 ~ 1.73)	0.80	0.01(77.3)	1.30(0.54 ~ 3.13)	0.56	0.10(56.5)	1.10(0.71 ~ 1.69)	0.67	0.01(79.2)
Colorectal	10	5552/7089	1.14(0.85 ~ 1.53)	0.39	0.02(53.5)	**0.92(0.85 ~ 0.99)**	**0.03**	**0.97(0.0)**	**0.94(0.87 ~ 1.01)**	**0.09**	**0.73(0.0)**	1.17(0.87 ~ 1.58)	0.29	0.02(54.0)	**0.97(0.91 ~ 1.03)**	**0.28**	**0.11(36.8)**
Lung	14	4767/8411	1.14(0.83 ~ 1.56)	0.42	0.02(49.3)	1.10(0.94 ~ 1.27)	0.24	0.00(63.4)	1.10(0.93 ~ 1.29)	0.27	0.00(71.9)	**0.97(0.80 ~ 1.17)**	**0.76**	**0.16(27.2)**	1.07(0.93 ~ 1.24)	0.36	0.00(74.1)
Head and neck	4	1035/1075	**1.28(0.81 ~ 2.02)**	**0.30**	**0.99(0.0)**	**0.84(0.70 ~ 1.01)**	**0.06**	**0.69(0.0)**	**0.88(0.73 ~ 1.05)**	**0.14**	**0.70(0.0)**	**1.35(0.85 ~ 2.13)**	**0.20**	**1.0(0.0)**	**0.94(0.81 ~ 1.01)**	**0.41**	**0.78(0.0)**
Breast cancer	3	1401/1423	**1.04(0.69 ~ 1.59)**	**0.85**	**0.49(0.0)**	**0.91(0.77 ~ 1.07)**	**0.04**	**0.66(0.0)**	**0.92(0.79 ~ 1.08)**	**0.30**	**0.67(0.0)**	**1.09(0.93 ~ 1.13)**	**0.70**	**0.49(0.0)**	**0.95(0.83 ~ 1.09)**	**0.44**	**0.66(0.0)**
**Source of control**																	
HB	16	5010/5638	**1.14(0.93 ~ 1.41)**	**0.21**	**0.99(0.0)**	**0.96(0.88 ~ 1.05)**	**0.37**	**0.20(22.7)**	**0.98(0.90 ~ 1.06)**	**0.63**	**0.32(11.4)**	**1.17(0.95 ~ 1.44)**	**0.15**	**0.99(0.0)**	**1.00(0.94 ~ 1.08)**	**0.94**	**0.66(0.0)**
PB	36	14191/21351	1.04(0.89 ~ 1.22)	0.61	0.01(39.4)	0.96(0.90 ~ 1.03)	0.22	0.01(39.4)	0.97(0.90 ~ 1.04)	0.40	0.00(50.7)	1.04(0.90 ~ 1.21)	0.56	0.05(30.0)	0.99(0.92 ~ 1.05)	0.64	0.00(57.2)
**Ethnicity**																	
Asian	13	2957/3770	**0.97(0.75 ~ 1.27)**	**0.84**	**0.51(0.0)**	**0.99(0.89 ~ 1.11)**	**0.90**	**0.26(18.6)**	**0.99(0.89 ~ 1.10)**	**0.88**	**0.14(30.6)**	**0.98(0.76 ~ 1.28)**	**0.90**	**0.59(0.0)**	1.01(0.90 ~ 1.13)	0.90	0.09(37.1)
Caucasian	32	12294/17831	1.10(0.93 ~ 1.30)	0.26	0.03(34.8)	0.95(0.88 ~ 1.02)	0.18	0.01(44.0)	0.97(0.89 ~ 1.04)	0.39	0.00(52.8)	**1.07(0.95 ~ 1.21)**	**0.27**	**0.11(24.4)**	0.99(0.92 ~ 1.06)	0.71	0.00(57.3)
Mixed	7	3921/5262	**0.90(0.73 ~ 1.12)**	**0.35**	**0.81(0.0)**	**0.94(0.86 ~ 1.04)**	**0.22**	**0.43(0.0)**	**0.94(0.86 ~ 1.03)**	**0.16**	**0.69(0.0)**	**0.92(0.74 ~ 1.14)**	**0.45**	**0.71(0.0)**	**0.95(0.88 ~ 1.02)**	**0.15**	**0.93(0.0)**
African	2	265/426	0.81(0.22 ~ 2.96)	0.75	0.08(21.3)	**1.09(0.79 ~ 1.50)**	**0.60**	**0.78(0.0)**	**1.06(0.78 ~ 1.45)**	**0.69**	**0.46(0.0)**	0.78(0.23 ~ 2.73)	0.70	0.08(66.6)	**1.03(0.94 ~ 1.01)**	**0.84**	**0.22(34.7)**
**Smoking status**																	
Smoker	9	2331/2542	1.08(0.64 ~ 1.83)	0.77	0.01(61.5)	1.19(0.88 ~ 1.60)	0.27	0.00(76.6)	1.16(0.85 ~ 1.58)	0.35	0.00(79.9)	1.04(0.68~1.60)	0.86	0.06(46.8)	1.09(0.84 ~ 1.41)	0.52	0.00(80.1)
Non-smoker	8	1498/2336	**1.25(0.90 ~ 1.75)**	**0.19**	**0.67(0.0)**	**1.05(0.91 ~ 1.21)**	**0.49**	**0.78(0.0)**	**1.08(0.94 ~ 1.23)**	**0.30**	**0.71(0.0)**	**1.24(0.89 ~ 1.72)**	**0.21**	**0.71(0.0)**	**1.08(0.96 ~ 1.22)**	**0.19**	**0.60(0.0)**

PB: population based; HB: hospital based. *P* ^*b*^: P-values for heterogeneity from Q test; I^2^ refers to the proportion of total variation owing to between-study heterogeneity.

Bold font marks where fixed effect model used.

**Figure 2:**
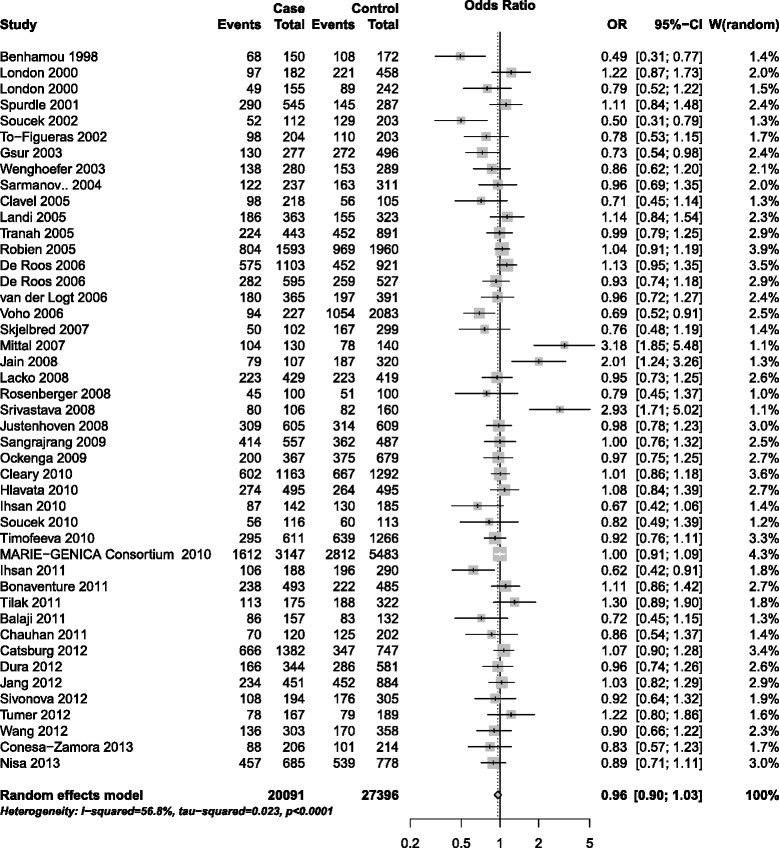
**Forest plot for association of EPHX1 Tyr113His polymorphism and cancer risk (dominant model, CT + CC vs. TT).**

For Tyr113His polymorphism, overall, no significantly elevated cancer risk could be observed in all genetic models (Table 1. homozygote model: OR = 1.05, 95% CI = 0.95–1.16; heterozygote model: OR = 0.94, 95% CI = 0.88–1.01; additive model: OR = 1.00, 95% CI = 0.95–1.05; dominant model: OR = 0.96, 95% CI = 0.90–1.03, Figure 2; recessive model: OR = 1.08, 95% CI = 0.99–1.18). When stratified by ethnicity, the significantly increased cancer risks were found among Asian population (homozygote model: OR = 1.46, 95% CI = 1.05–2.03; recessive model: OR = 1.39, 95% CI = 1.10–1.76) and Mixed population (homozygote model: OR = 1.17, 95% CI = 1.02–1.34; recessive model: OR = 1.17, 95% CI = 1.02–1.33). Stratified analyses by cancer types, smoking status and source of controls indicated no evidence of significant association between Tyr113His polymorphism and the cancer risk. Furthermore, individuals carrying Tyr/His or His/His genotype have a significantly reduced risk of lung cancer (heterozygote model: OR = 0.80, 95% CI = 0.65-0.98; dominant model: OR = 0.81, 95% CI = 0.68–0.98).

**Figure 3:**
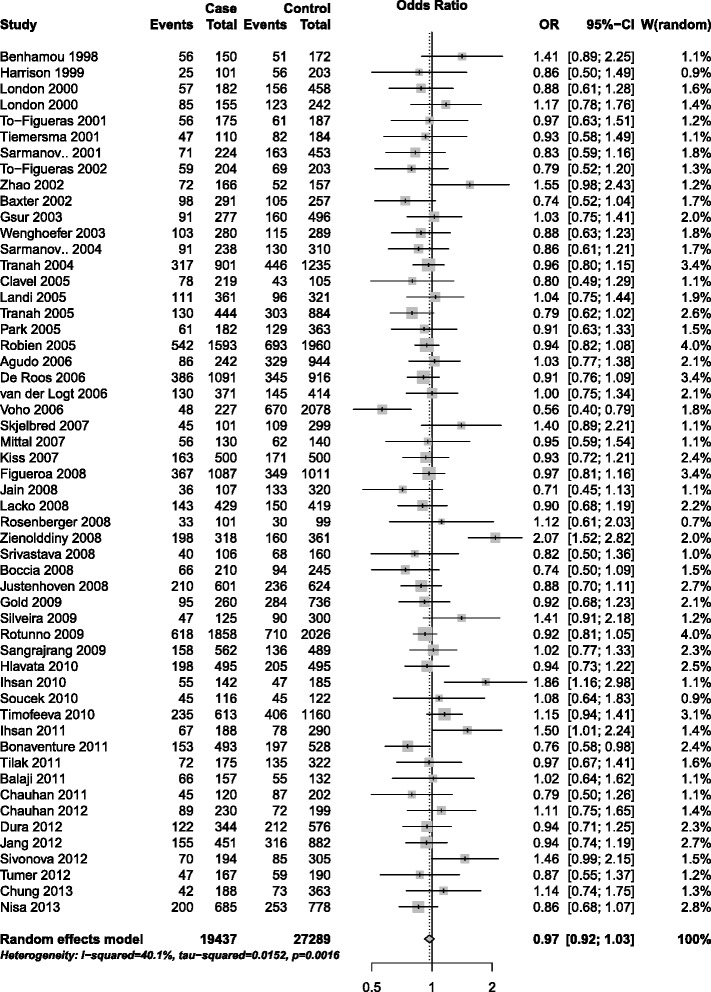
**Forest plot for association of EPHX1 His139Arg polymorphism and cancer risk (dominant model, GA + GG vs. AA).**

With respect to His139Arg polymorphism, similarly, the combined results did not show any association with the elevated risk of cancer for all genetic models (homozygote model: OR = 1.05, 95% CI = 0.93–1.18; heterozygote model: OR = 0.96; 95% CI = 0.91–1.01; additive model: OR = 0.99, 95% CI = 0.94–1.04; dominant model: OR = 0.97, 95% CI = 0.92–1.03, Figure 3; recessive model: OR = 1.02, 95% CI = 0.93–1.13). When stratified according to cancer types, no significant association with increased cancer risk was demonstrated in all subgroups for overall population. However, the result suggested a decreased risk for blood cancers (additive model: OR = 0.91, 95% CI = 0.83–0.99; dominant model: OR = 0.90, 95% CI = 0.81–0.99) and colorectal cancer (heterozygote model: OR = 0.92; 95% CI = 0.85–0.99). In the subgroup analysis by source of controls, smoking status and ethnicity, no significant association with cancer risk was observed in all subgroups (Table 2).

### Meta-regression and sensitivity analyses

Heterogeneity is a potential issue that may affect the interpretation of the results. As for Tyr113His polymorphism, the heterogeneity was observed in all genetic models and the detailed data are shown in Table 1. With respect to His139Arg polymorphism (Table 2), the heterogeneity was detected in homozygote comparison, heterozygote model, dominant model and additive model. We therefore explored the source of heterogeneity by cancer type, ethnicity, and source of control by meta-regression in all comparisons with significant heterogeneity. As a result, for Tyr113His polymorphism, source of control may be the major source of heterogeneity in homozygote model (P = 0.024), heterozygote model (P = 0.000), dominant model (P = 0.002) and additive model (P = 0.005), but not recessive model. However, for His139Arg polymorphism, none of these variables showed statistically significant associations in multivariate meta-regression model (P > 0.05), suggesting factors mentioned above could not explain the heterogeneity among studies.

The influences of each individual study on the overall ORs for Tyr113His/His139Arg polymorphisms were evaluated. The results showed the pooled ORs of these two polymorphisms were not materially altered by the omission of any individual study, suggesting credibility for the conclusions (Additional file 4: Figure S1 and Additional file 5: Figure S2).

### Publication bias

**Figure 4:**
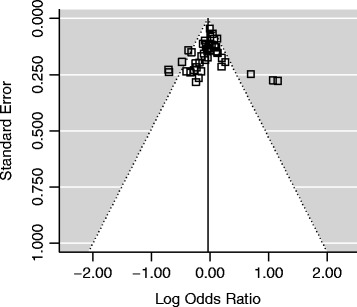
**Begg’s funnel plot for publication bias in studies on EPHX1 Tyr113His polymorphism and cancer (dominant model, CT + CC vs. TT).**

**Figure 5:**
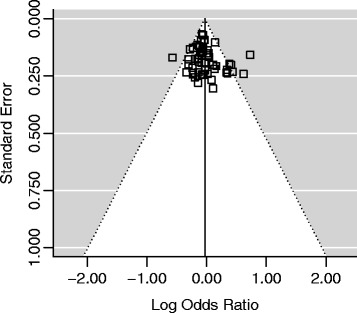
**Begg’s funnel plot for publication bias in studies on EPHX1 His139Arg polymorphism and cancer (dominant model, GA + GG vs. AA).**

Begg’s funnel plot and Egger’s test were performed to assess the publication bias of literatures. The shape of funnel plots (Figures 4 and 5) did not reveal any evidence of asymmetry. The statistical results of Egger’s test still did not show publication bias for Tyr113His polymorphism (additive model: P = 0.146; homozygote comparison: P = 0.620; heterozygote model: P = 0.189; dominant model: P = 0.054; recessive model: P = 0.915) and His139Arg polymorphism (additive model: P = 0.125; homozygote comparison: P = 0.847; heterozygote model: P = 0.255; dominant model: P = 0.111; recessive model: P = 0.153).

## Discussion

With increased knowledge of human gene functions and the architecture of genetic variations, it has become clear that individual variation in genetic backgrounds, such as single nucleotide polymorphism, could substantially influence cancer risk with specific environmental exposure. However, evidences from studies of genetic epidemiology were usually too conflicting to draw conclusions. Meta-analysis shed light on objective and comprehensive assessment of the associations between polymorphisms and cancer risks. Several single nucleotide polymorphisms were identified as cancer risk factors for specific populations by means of meta-analysis [82-84].

The genetic polymorphisms of EPHX1, Tyr113His and His139Arg, may affect enzyme activity involved in general oxidative defenses against a number of environmental substances [6]. Variations in the expression and activity level of EPHX1 as a result of such polymorphisms could cause individual variations of detoxifying capability, then further influence the risk of chemical carcinogen-induced cancers [85]. The findings from some previous studies suggested that genetic polymorphism in EPHX1 has important roles in the development of cancers [55,86-88]. However, others reported no association of EPHX1 polymorphisms with risk of cancers [24,26,34,45]. This inconsistency may be due to tremendous difference in sample size, diverse ethnic background, sampling bias, publication bias, or inadequate statistical power. The benefits of meta-analysis include a larger number of participants, different geographic locations, and the possibility of inclusion of a wider range of population groups, all of which could derive a more precise estimation and further increase the generalizability of the results.

In this meta-analysis, 99 eligible case–control studies including 39,528 cases and 54,685 controls were included to provide a comprehensive assessment of the relationship between EPHX1 polymorphisms and cancer risk. The results revealed that neither EPHX1 Tyr113His polymorphism, nor His139Arg polymorphism have significant association with the cancer susceptibility for all comparing models when all studies were accumulated together. Further stratified analysis according to cancer types, smoking status, or source of controls did not suggest a significantly increased risk. Moreover, His139Arg polymorphism might play a potentially protective role in the development of blood cancer based on dominant model and additive model, and colorectal cancer by heterozygous model. Meanwhile Tyr113His polymorphism showed possible protective effect on risk for lung cancer by heterozygote model and dominant model.

Stratified analysis by ethnicity allowed for assessing the ethnic differences in the association of cancer risk. As for His139Arg polymorphism, no significant associations were found in any genetic model among all populations. However, with respect to the Tyr113His polymorphism, an increased risk of cancer based on homozygote and recessive model could be observed in Asian and mixed population, indicating there is an obvious race-specific effect in the association. It was consistent with the results from two recent meta-analyses for hepatocellular cancer [89] and lung cancer [90]. Further subgroup analysis by the cancer types in Asian and Mixed population was not performed due to the limited data for individual cancer type according to our inclusion criteria.

Heterogeneity between studies should be noted because it may potentially affect the strengths of the meta-analysis. In the current meta-analysis, significance heterogeneity was observed for both EPHX1 Tyr113His and His139Arg polymorphisms. Thus, random-effect models were used if significant heterogeneity was identified. Furthermore, multivariate meta-regression analysis involving covariates, such as source of control, cancer type, ethnicity, was performed to explore the source of heterogeneity. The results from meta-regression emphasized that the heterogeneity of polymorphism Tyr113His was associated with source of control in homozygote model, heterozygote model, dominant model and additive model, but not recessive model. Neither cancer type, nor ethnicity was found to be the source of heterogeneity. However, as for His139Arg, results indicated none of these three covariates could be the main source of the between-study heterogeneity. It suggested that some other confounding factors, such as environmental exposures, gene-gene interaction, and lifestyle might lead to the heterogeneity. Large studies for both polymorphisms with comprehensive classification information are needed to facilitate the subgroup analysis according to these factors, which is unavailable for present meta-analysis because of inadequate information from our original data sources.

Although our result is suggestive, there are still some limitations inherited from the published studies and our analysis strategies. First, the present conclusion was drawn based on unadjusted estimates, while a more precise analysis with the necessary adjustment by other covariates including age, lifestyle, gene–gene interactions and environmental factors should be conducted when more detailed individual data were available. Second, although some results were significant, the p–values were on the borderline, i.e. slightly less than 0.05. Further large and well-designed studies are required for confirmation. Finally, most studies were from Caucasian population, it is critical that larger and well-designed multi-centric studies based on Asians and other racial-ethnic groups should be performed to re-evaluate the association. In spite of these, our meta-analysis also had some advantages. First, the quality of case–control studies included in current meta-analysis was satisfactory and met our inclusion criterion, ensuring the quality of our results. Second, the sensitivity analysis showed that no individual study materially altered the pooled ORs indicating statistical stableness and robustness of the current meta-analysis. In addition, no publication bias for the association between these two polymorphisms and cancer risk could be observed, which further confirmed the credibility.

In conclusion, our investigations suggested that the EPHX1 His139Arg polymorphism might not contribute to the susceptibility of all cancer types for overall population, whereas Tyr113His polymorphism might be associated with increased risk of cancer in the Asian and mixed population. Larger well-designed epidemiological studies with different cancer types, ethnically diverse populations and functional evaluations are warranted to confirm our findings.

## Additional files

Additional file 1: Table S1. PRISMA Checklist for this meta-analysis. Additional file 2: Table S2. Principal characteristics of the studies included on EPHX1 Tyr113His polymorphism. Additional file 3: Table S3. Principal characteristics of the studies included on EPHX1 His139Arg polymorphism. Additional file 4: Figure S1. Sensitivity analysis of the summary OR of the association between EPHX1 Tyr113His polymorphism and cancer susceptibility in dominant model. Results were computed by omitting each study in turn. Random-effects model was used. The two ends of the dotted lines represent the 95% confidence interval. Additional file 5: Figure S2. Sensitivity analysis of the summary OR of the association between EPHX1 His139Arg polymorphism and cancer susceptibility in dominant model. Results were computed by omitting each study in turn. Random-effects model was used. The two ends of the dotted lines represent the 95% confidence interval.

## Abbreviations

HWE: Hardy–Weinberg equilibrium
EPHX1: Epoxide hydrolase 1
SNP: Single nucleotide polymorphism
OR: Odds ratio
CI: Confidence interval
