# Supplementary material for: Quantitative assessment of the influence of EPHX1 gene polymorphisms and cancer risk: a meta-analysis with 94,213 subjects
Source: J Exp Clin Cancer Res. 2014 Sep 28;33(1):82. doi: 10.1186/s13046-014-0082-9 (PMC4189664; doi:10.1186/s13046-014-0082-9)
Supplement: Additional file 2: Table S2. — Principal characteristics of the studies included on EPHX1 Tyr113His polymorphism. [file 13046_2014_82_MOESM2_ESM.pdf]

Table S2. Principal characteristics of the studies included on EPHX1 Tyr113His polymorphism.

First Author	Year	Country	Ethnicity	Cancer Type	Source of Control	Genotypes distribution (case/control)			HWE
						TT	TC	CC	
Conesa-Zamora	2013	Spain	Caucasian	lymphoma	PB	118/113	71/83	17/18	0.62
Catsburg	2012	USA	Mixed	prostate cancer	PB	716/400	541/295	125/52	0.81
Dura	2012	Holland	Caucasian	esophageal cancer	PB	178/295	136/228	30/58	0.16
Nisa	2013	Japan	Asian	colorectal cancer	PB	228/239	342/396	115/143	0.35
Jang	2012	Canada	Mixed	pancreatic cancer	PB	217/432	192/374	42/78	0.82
Ihsan	2011	India	Asian	lung cancer	HB	82/94	51/133	55/63	0.22
Bonaventure	2011	France	Caucasian	childhood acute leukemia	PB	255/263	197/190	41/32	0.77
Sivonova	2012	Slovak	Caucasian	prostate cancer	PB	86/129	94/140	14/36	0.83
Tumer	2012	Turkey	Asian	childhood ALL	PB	89/110	47/62	31/17	0.06
Tilak	2011	India	Asian	lung cancer	PB	62/134	85/157	28/31	0.12
Wang	2012	USA	Mixed	colorectal cancer	FB	167/188	108/141	28/29	0.72
Balaji	2011	India	Asian	oral cancer	HB	71/49	61/64	25/19	0.8
Cleary	2010	Canada	Caucasian	colorectal cancer	PB	561/625	502/549	100/118	0.87
Hlavata	2010	Czech	Caucasian	colorectal cancer	HB	221/231	224/212	50/52	0.75
Chauhan	2011	India	Asian	Adult AML	PB	50/77	48/85	22/40	0.07
Ihsan	2010	India	Asian	esophageal cancer	PB	55/55	49/96	38/34	0.48
Soucek	2010	Czech	Caucasian	HNC	PB	60/53	42/48	14/12	0.82
Timofeeva	2010	Germany	Caucasian	lung cancer	PB	316/627	238/520	57/119	0.46
MARIE-GENICA Consortium	2010	Germany	Caucasian	breast cancer	PB	1535/2671	1345/2322	267/490	0.65
Sangrajrang	2009	Thailand	Asian	breast cancer	HB	143/125	286/247	128/115	0.74
Ockenga	2009	Germany	Caucasian	pancreatic cancer	PB	167/304	164/311	36/64	0.22
Jain	2008	India	Asian	esophageal cancer	PB	28/133	66/156	13/31	0.13
Lacko	2008	Holland	Caucasian	HNC	PB	206/196	180/185	43/38	0.55
Rosenberger	2008	Germany	Caucasian	lung cancer	PB	55/49	38/43	7/8	0.74
Srivastava	2008	India	Asian	bladder cancer	unknown	26/78	57/64	23/18	0.38
Skjelbred	2007	Norway	Caucasian	colorectal cancer	PB	52/132	41/134	9/33	0.91
Mittal	2007	India	Asian	prostate cancer	unknown	26/62	67/60	37/18	0.56
Justenhoven	2008	Germany	Caucasian	breast cancer	PB	296/295	246/269	63/45	0.12
De Roos	2006	USA	Mixed	NHL	PB	528/469	471/383	104/69	0.45
De Roos	2006	USA	Mixed	brain cancer	HB	313/268	213/216	69/43	0.95
Clavel	2005	France	Mixed	childhood leukemia	HB	120/49	73/39	25/17	0.06
van der Logt	2006	Holland	Caucasian	colorectal cancer	PB	185/194	141/165	39/32	0.71
Landi	2005	Spain	Caucasian	colorectal cancer	HB	177/168	153/126	33/29	0.45
Voho	2006	Finland	Caucasian	lung cancer	PB	133/1029	81/865	13/189	0.71
Tranah	2005	USA	Caucasian	colorectal cancer	PB	219/439	190/371	34/81	0.84
Robien	2005	USA	Mixed	colorectal cancer	PB	789/991	655/795	149/174	0.42

Sarmanová	2004	Czech	Caucasian	breast cancer	HB	115/148	77/124	45/39	0.11
Gsur	2003	Austria	Caucasian	lung cancer	HB	147/224	114/218	16/54	0.93
Wenghoefer	2003	Germany	Caucasian	HNC	HB	142/136	112/128	26/25	0.51
Soucek	2002	Czech	Caucasian	lymphoma	PB	60/74	33/90	19/39	0.22
To-Figueras	2002	Spain	Caucasian	laryngeal cancer	PB	106/93	83/94	15/16	0.24
Spurdle	2001	Australia	Caucasian	ovarian cancer	PB	255/142	233/114	57/31	0.26
London	2000	USA	Caucasian	lung cancer	PB	85/237	82/184	15/37	0.88
London	2000	USA	African	lung cancer	PB	106/153	48/77	1/12	0.57
Benhamou	1998	France	Caucasian	lung cancer	HB	82/64	46/77	22/31	0.26

PB: population based; HB: hospital based; FB: family based

HWE: Hardy-Weinberg equilibrium (significant at the 0.05 level)

ALL: acute lymphoblastic leukemia; AML: acute myelocytic leukemia; HNC: head and neck cancer; NHL: Non Hodgkin lymphoma.
