# Supplementary material for: Quantitative assessment of the influence of EPHX1 gene polymorphisms and cancer risk: a meta-analysis with 94,213 subjects
Source: J Exp Clin Cancer Res. 2014 Sep 28;33(1):82. doi: 10.1186/s13046-014-0082-9 (PMC4189664; doi:10.1186/s13046-014-0082-9)
Supplement: Additional file 3: Table S3. — Principal characteristics of the studies included on EPHX1 His139Arg polymorphism. [file 13046_2014_82_MOESM3_ESM.pdf]

Table S3. Principal characteristics of the studies included on EPHX1 His139Arg polymorphism.

First Author	Year	Country	Ethnicity	Cancer Type	Source of Control	Genotypes distribution (case/control)			HWE
						AA	AG	GG	
Chung	2013	China	Asian	urothelial cancer	HB	146/290	39/66	3/7	0.17
Chauhan	2012	India	Asian	acute leukemia	PB	141/127	80/64	9/8	0.99
Dura	2012	Holland	Caucasian	esophageal cancer	PB	222/364	111/187	11/25	0.87
Nisa	2013	Japan	Asian	colorectal cancer	PB	485/525	182/224	18/29	0.41
Jang	2012	Canada	Mixed	pancreatic cancer	PB	296/566	135/280	20/36	0.85
Ihsan	2011	India	Asian	lung cancer	HB	121/212	59/70	8/8	0.45
Bonaventure	2011	France	Caucasian	childhood acute leukemia	PB	340/331	135/178	18/19	0.41
Sivonova	2012	Slovak	Caucasian	prostate cancer	PB	124/220	62/76	8/9	0.44
Tumer	2012	Turkey	Asian	childhood ALL	PB	120/131	43/55	4/4	0.52
Tilak	2011	India	Asian	lung cancer	PB	103/187	62/117	10/18	0.96
Balaji	2011	India	Asian	oral cancer	HB	91/77	58/50	8/5	0.37
Hlavata	2010	Czech	Caucasian	colorectal cancer	HB	297/290	173/183	25/22	0.31
Chauhan	2011	India	Asian	Adult AML	PB	75/115	38/78	7/9	0.35
Ihsan	2010	India	Asian	esophageal cancer	PB	87/138	48/45	7/2	0.43
Soucek	2010	Czech	Caucasian	HNC	PB	71/77	40/41	5/4	0.6
Timofeeva	2010	Germany	Caucasian	lung cancer	PB	378/754	212/361	23/45	0.83
Gold	2009	USA	Mixed	multiple myeloma	PB	165/452	84/248	11/36	0.79
Silveira	2009	Brazil	Mixed	ALL	HB	78/210	44/77	3/13	0.09
Rotunno	2009	Italy	Caucasian	lung cancer	PB	1240/1316	567/628	51/82	0.52
Sangrajrang	2009	Thailand	Asian	breast cancer	HB	404/353	147/128	11/8	0.35
Figueroa	2008	Spain	Caucasian	bladder cancer	HB	720/662	319/312	48/37	0.97
Jain	2008	India	Asian	esophageal cancer	PB	71/187	29/116	7/17	0.86
Lacko	2008	Holland	Caucasian	HNC	PB	286/269	123/135	20/15	0.7
Rosenberger	2008	Germany	Caucasian	lung cancer	PB	68/69	29/27	4/3	0.86
Zienolddiny	2008	Norway	Caucasian	lung cancer	PB	120/201	153/128	45/32	0.08
Srivastava	2008	India	Asian	bladder cancer	unknown	66/92	36/55	4/13	0.25
Skjelbred	2007	Norway	Caucasian	colorectal cancer	PB	56/190	27/90	18/19	0.07
Mittal	2007	India	Asian	prostate cancer	unknown	74/78	49/49	7/13	0.2
Kiss	2007	Hungary	Caucasian	colorectal cancer	PB	337/329	157/161	6/10	0.05
Boccia	2008	Italy	Caucasian	HNC	HB	144/151	59/88	7/6	0.1
Justenhoven	2008	Germany	Caucasian	breast cancer	PB	391/388	182/213	28/23	0.35
Agudo	2006	EPIC study	Caucasian	gastric cancer	PB	156/615	76/289	10/40	0.41
De Roos	2006	USA	Mixed	NHL	PB	705/571	345/304	41/41	0.95
Clavel	2005	France	Mixed	childhood leukemia	HB	141/62	67/41	11/2	0.1
van der Logt	2006	Holland	Caucasian	colorectal cancer	PB	241/269	106/128	24/17	0.72
Landi	2005	Spain	Caucasian	colorectal cancer	HB	250/225	98/85	13/11	0.4

Voho	2006	Finland	Caucasian	lung cancer	PB	179/1408	48/608	0/62	0.71
Tranah	2005	USA	Caucasian	colorectal cancer	PB	314/581	117/273	13/30	0.76
Park	2005	USA	Mixed	lung cancer	HB	121/234	54/118	7/11	0.4
Robien	2005	USA	Mixed	colorectal cancer	PB	1051/1267	479/605	63/88	0.15
Sarmanová	2004	Czech	Caucasian	breast cancer	HB	147/180	83/115	8/15	0.54
Tranah	2004	USA	Caucasian	colorectal cancer	PB	584/789	275/406	42/40	0.16
Gsur	2003	Austria	Caucasian	lung cancer	HB	186/336	80/144	11/16	0.91
Wenghoefer	2003	Germany	Caucasian	HNC	HB	177/174	91/106	12/9	0.13
To-Figueras	2002	Spain	Caucasian	laryngeal cancer	PB	145/134	56/62	3/7	0.96
Zhao	2002	USA	Caucasian	lung cancer	PB	94/105	62/46	10/6	0.73
Baxter	2002	Australia	Caucasian	ovarian cancer	HB	193/152	82/92	16/13	0.85
To-Figueras	2001	Spain	Caucasian	lung cancer	PB	119/126	53/54	3/7	0.69
Tiemersma	2001	Sudan	African	hepatocellular cancer	PB	63/102	44/69	3/13	0.78
Sarmanová	2001	Czech	Caucasian	lymphoma	PB	153/290	64/140	7/23	0.26
London	2000	USA	Caucasian	lung cancer	PB	125/302	50/136	7/20	0.35
London	2000	USA	African	lung cancer	PB	70/119	70/105	15/18	0.43
Harrison	1999	UK	Caucasian	colorectal cancer	PB	76/147	21/53	4/3	0.47
Benhamou	1998	France	Caucasian	lung cancer	HB	94/121	53/49	3/2	0.22

EPIC study: Denmark, France, Germany, Greece, Italy, the Netherlands, Norway, Spain, Sweden, and the United Kingdom.

PB: population based; HB: hospital based.

HWE: Hardy-Weinberg equilibrium (significant at the 0.05 level).

ALL: acute lymphoblastic leukemia; AML: acute myelocytic leukemia; HNC: head and neck cancer; NHL: Non Hodgkin lymphoma.
