# Supplementary figures and images for: Quantitative assessment of the influence of EPHX1 gene polymorphisms and cancer risk: a meta-analysis with 94,213 subjects
Source: J Exp Clin Cancer Res. 2014 Sep 28;33(1):82. doi: 10.1186/s13046-014-0082-9 (PMC4189664; doi:10.1186/s13046-014-0082-9)

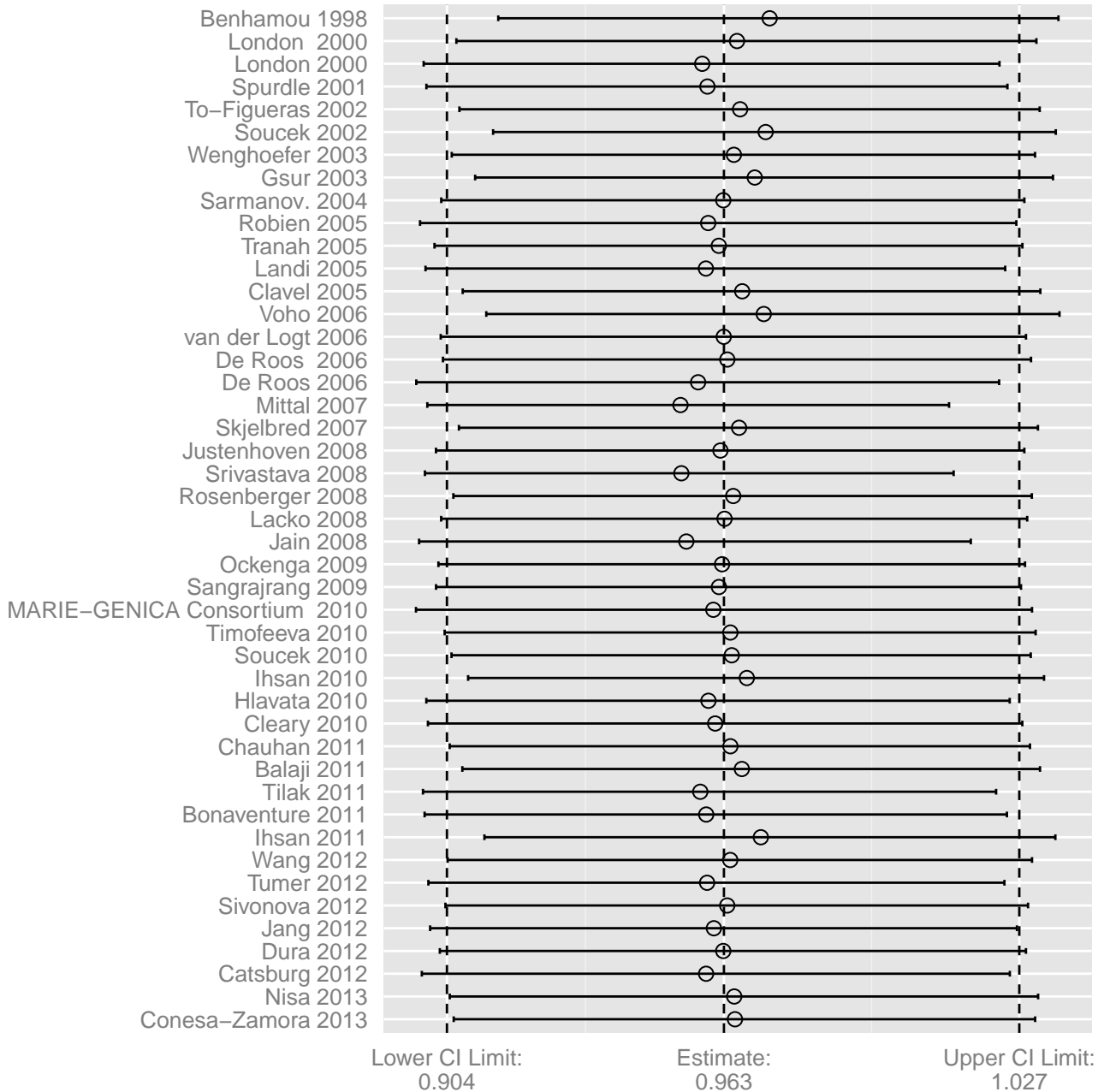

Supplement: Additional file 4: Figure S1. — Sensitivity analysis of the summary OR of the association between EPHX1 Tyr113His polymorphism and cancer susceptibility in dominant model. Results were computed by omitting each study in turn. Random-effects model was used. The two ends of the dotted lines represent the 95% confidence interval. [file 13046_2014_82_MOESM4_ESM.pdf]

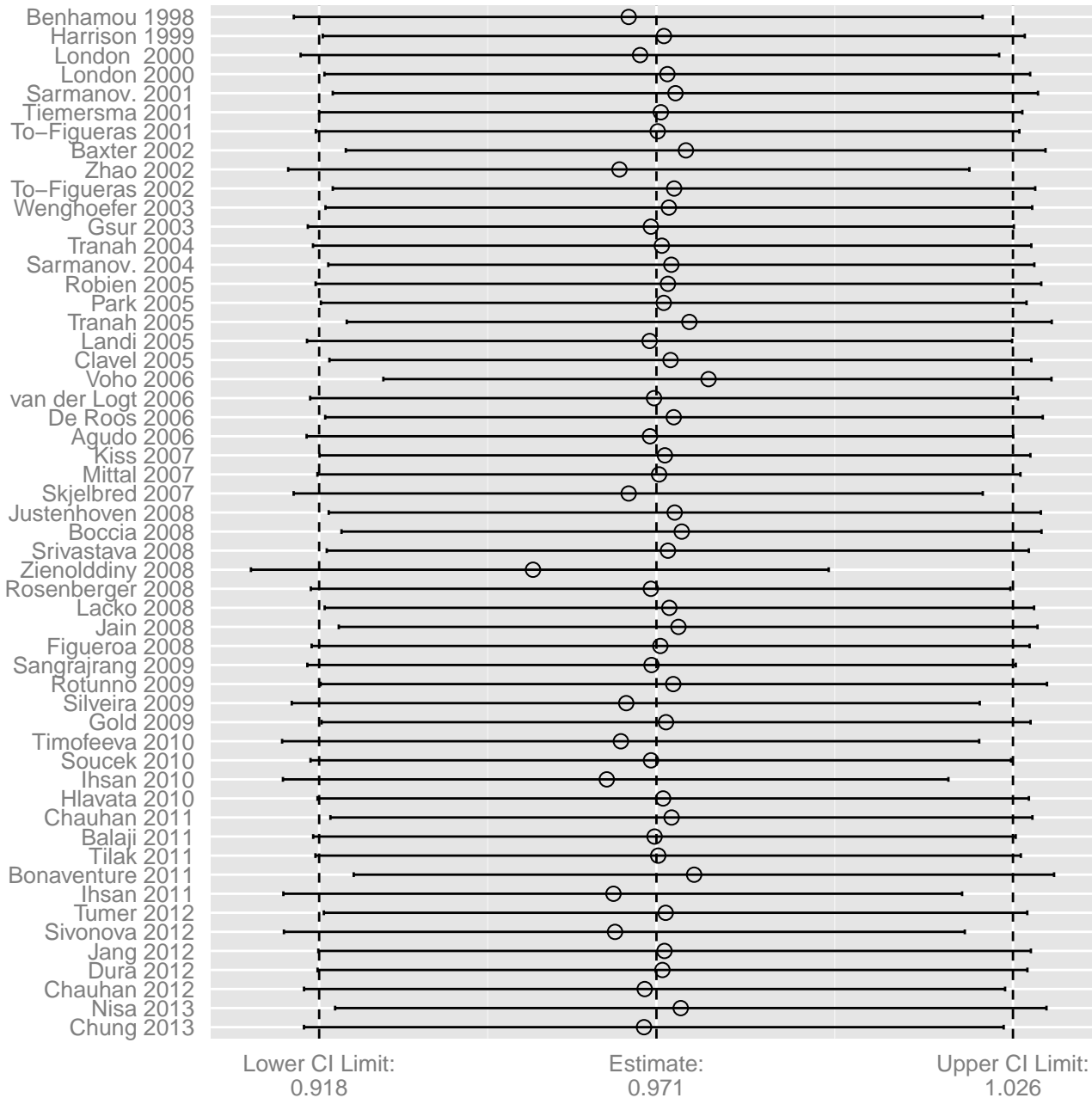

Supplement: Additional file 5: Figure S2. — Sensitivity analysis of the summary OR of the association between EPHX1 His139Arg polymorphism and cancer susceptibility in dominant model. Results were computed by omitting each study in turn. Random-effects model was used. The two ends of the dotted lines represent the 95% confidence interval. [file 13046_2014_82_MOESM5_ESM.pdf]
